# Documenting the Need for Patient-Centered Relevant Outcomes in Adult Day Services

**DOI:** 10.1093/geroni/igab046.322

**Published:** 2021-12-17

**Authors:** Jonelle Boafo, Keith Anderson, Abraham Brody, Tina Sadarangani

**Affiliations:** 1 Rory Meyers College of Nursing, New York, New York, United States; 2 University of Texas at Arlington, Arlington, Texas, United States; 3 NYU Hartford Institute for Geriatric Nursing, New York, New York, United States; 4 New York University Rory Meyers College of Nursing, New York University Rory Meyers College of Nursing, New York, United States

## Abstract

ADCs are not uniformly regulated at the federal or state level, resulting in the absence of uniform data collection. The lack of large-scale data has resulted in a dearth of evidence on the role ADC services play in the health and well-being of their clients, particularly persons living with dementia (PLWD). The purpose of this study was to compare data being collected across states and evaluate the degree to which patient centered relevant outcomes (PCROs) are being collected. A review of ADC regulations in 50 states found that <10 states, required standardized reporting on ADC participants. Regulatory forms relied on clinical judgment as opposed to validated tools, and focused on eligibility for services as opposed to independence, engagement, or clinical interventions in the ADC. Emphasizing collection of PCROs in ADCs, beginning at the state level, is an essential step in documenting the value and effectiveness of ADCs, particularly for PLWD.

